# Chemotherapy-induced weight gain in early-stage breast cancer: a prospective matched cohort study reveals associations with inflammation and gut dysbiosis

**DOI:** 10.1186/s12916-023-02751-8

**Published:** 2023-05-11

**Authors:** John Walker, Anil Abraham Joy, Larissa J. Vos, Trevor H. Stenson, John R. Mackey, Juan Jovel, Dina Kao, Karen L. Madsen, Gane Ka-Shu Wong

**Affiliations:** 1 Department of Oncology, Faculty of Medicine and Dentistry, University of Alberta, Edmonton, AB T6G 2H5 Canada; 2 Division of Medical Oncology, Cross Cancer Institute, Edmonton, AB T6G 1Z2 Canada; 3 Clinical Trials Unit, Cross Cancer Institute, Edmonton, AB T6G 1Z2 Canada; 4 The Applied Genomics Core (TAGC), Faculty of Medicine, University of Alberta, Edmonton, AB T6G 2X8 Canada; 5 Division of Gastroenterology, Department of Medicine, Faculty of Medicine and Dentistry, University of Alberta, Edmonton, AB T6G 2H5 Canada; 6 Department of Biological Sciences, Faculty of Science, University of Alberta, Edmonton, AB T6G 2E9 Canada

**Keywords:** Adjuvant chemotherapy, Gastrointestinal dysbiosis, Inflammatory biomarkers, Inflammatory bowel disease, Dual-energy X-ray absorptiometry, Weight gain

## Abstract

**Background:**

Early-stage breast cancer patients treated with chemotherapy risk the development of metabolic disease and weight gain, which can result in increased morbidity and reduced quality of life in survivorship. We aimed to analyze changes within the gastrointestinal microbiome of early-stage breast cancer patients treated with and without chemotherapy to investigate a potential relationship between dysbiosis, a systemic inflammatory response, and resultant anthropomorphic changes.

**Methods:**

We undertook an a priori analysis of serially collected stool and plasma samples from 40 patients with early-stage breast cancer who underwent adjuvant endocrine therapy only, adjuvant chemotherapy only, or both. Gut microbiota were assessed by metagenomic comparison of stool samples following deep sequencing. Inflammatory biomarkers were evaluated by proteomic analysis of plasma and measurement of fecal calprotectin. Body composition was investigated by dual-energy X-ray absorptiometry to determine biomass indices.

**Results:**

As opposed to treatment with endocrine therapy only, chemotherapy resulted in statistically and clinically significant weight gain and an increase in the android to gynoid ratio of fat distribution. Patients treated with chemotherapy gained an average of 0.15% total mass per month, as opposed to a significantly different loss of 0.19% in those patients who received endocrine-only therapy. Concurrently, a twofold increase in fecal calprotectin occurred after chemotherapy that is indicative of interferon-dependent inflammation and evidence of colonic inflammation. These anthropomorphic and inflammatory changes occurred in concert with a chemotherapy-dependent effect on the gut microbiome as evidenced by a reduction in both the abundance and variety of microbial species.

**Conclusions:**

We confirm the association of chemotherapy treatment with weight gain and potential deleterious anthropometric changes and suggest that alterations of bacterial flora may contribute to these phenomena through the induction of systemic inflammation. Consequently, the gut microbiome may be a future target for intervention in preventing chemotherapy-dependent anthropometric changes.

**Supplementary Information:**

The online version contains supplementary material available at 10.1186/s12916-023-02751-8.

## Background

Improvements in treatment outcomes for individuals with early-stage breast cancer mark a significant achievement in cancer care. Patients with localized disease or regional nodal metastases now experience 5-year survival rates of 99% and 85%, respectively [1]. Advances in the biological understanding of the disease have improved care for these patients with the development of increasingly effective systemic therapies that reduce the risk of distant recurrence by treating subclinical residual or metastatic disease. In early studies when compared to observation, adjuvant chemotherapy reduced the risk of disease-specific mortality by nearly 30% [2] with modern regimens further reducing mortality [3].

As treatment outcomes for early-stage breast cancer improved, attention turned to reducing short-term and long-term treatment-associated morbidity. Cancer survivorship examines the field of care and research pertaining to people either living with or cured of their disease. Chemotherapy-induced syndromes such as fatigue, anxiety, depressive symptoms, and peripheral neuropathy are relatively common among survivors of breast cancer. More recently, alterations in metabolic function have also been described [4] and among breast cancer patients weight gain is a common side effect that decreases the quality of life while increasing the risk of diabetes, hypertension, and resultant cardiovascular risk [5]. Weight gain following an early-stage breast cancer diagnosis has also been associated with an increase in all-cause mortality [6, 7]. Factors associated with chemotherapy-associated weight gain include patient age, menopausal status, and reduced physical activity, while most studies do not find a simple correlative relationship between increased caloric intake and weight gain [8, 9].

The role of intestinal microbiota has also garnered recent attention as an influence on health and disease outcomes [10]. The human gut microbiome is composed of a multitude of bacteria with more than 500 characterized species classified within four dominant phyla: Firmicutes, Bacteroidetes, Actinobacteria, and Proteobacteria [11]. The gut microbiota has been shown to differ from healthy controls, a state known as dysbiosis, in a number of chronic diseases including atherosclerotic cardiovascular disease [12], type 2 diabetes [13], and obesity [14]. Dysbiosis within the gut microbiome and the relationship to inflammation is well described in the setting of inflammatory bowel disease (IBD) where reduced bacterial diversity has shown a strong association with active disease. Ott et al. demonstrated a reduction of species diversity within the gut of 30% and 50% when ulcerative colitis and Crohn’s disease patients were compared against non-inflamed controls [15]. The opposite also appears true; restoration of gut microbial diversity after fecal-microbial transplantation ameliorates disease in chronic Clostridium difficile infection [16] as well as IBD [17]. As our knowledge of the relationship between the microbiome and human health has grown, it has become increasingly apparent that dysbiosis within this complex and dynamic ecosystem may have a myriad of clinical consequences.

While the intended target of cytotoxic chemotherapy is the replicative machinery involved with cellular division in cancer, some chemotherapies are derived from antibiotics, and as such, they may also have unintended antimicrobial effects. For instance, anthracyclines act mainly through the intercalation of deoxyribonucleic acid (DNA), which in turn inhibits DNA synthesis and ribonucleic acid (RNA) production. Taxane chemotherapies, disrupt microtubule function thereby preventing mitotic depolymerization and thus inhibiting cellular division. Both of these agents are mainstays in the treatment of early-stage breast cancer, are administered systemically, and may affect the microbiota within the gut. Hepatobiliary secretion in feces is the predominant route of elimination for taxane and anthracycline chemotherapies and a recent report suggests significant bactericidal activity associated with both agents [18].

Perturbation of the gut flora in breast cancer patients receiving chemotherapy is known to occur [19], but whether weight gain reflects clinically significant changes to body composition, and the mechanisms by which this occurs are not known. We hypothesized that cytotoxic chemotherapy in women with early-stage breast cancer could affect the gut microbiota, modify host metabolism relevant to inflammation, and result in weight gain and deleterious anthropometric changes. To investigate this possibility, we undertook a prospective, controlled, matched-cohort study of women with early-stage breast cancer to characterize changes in body weight and composition in conjunction with the analysis of temporal changes in gut microbiota and inflammatory biomarkers.

## Methods

### Study design and population

Our patient population was screened for enrolment in this prospective study between the years 2016 and 2019. Study eligibility criteria included age ≥ 18 years; histologically or cytologically confirmed, early-stage invasive breast cancer, eligible for definitive breast surgery; and ability to sign informed consent and comply with study procedures. Exclusion criteria included antibiotic therapy within two weeks of enrolment; uncontrolled systemic infection; history of chronic diarrhea, gastroenteritis, or other active IBD; known HIV positivity; and presence of concurrent active malignancy or other severe diseases. We utilized a matched-cohort study design with the intent of enrolling similar populations of patients who in addition to surgery received treatment with (i) chemotherapy as an adjuvant treatment to surgery, (ii) endocrine therapy only following surgery, or (iii) the combination of both given sequentially. Potential participants were recruited from the outpatient department of a tertiary cancer center, the Cross Cancer Institute in Edmonton, Alberta, Canada. After obtaining informed consent, serial stool, urine, and blood samples were collected from breast cancer patients pre-operatively, before and after adjuvant systemic therapy (chemotherapy, endocrine, or both), and 1 year following enrolment to study. Relevant past and current medical information were simultaneously collected from the patients’ medical records. This study was conducted at the Cross Cancer Institute, an accredited, tertiary cancer care center and a Good Clinical Practice (GCP)-compliant research facility.

### DXA body composition analysis

To measure total fat and lean body mass, full-body dual-energy X-ray absorptiometry (DXA) scans were obtained using Lunar Prodigy DXA and enCORE software, version 10.50 (GE Healthcare, Madison, WI, USA). These exams were reviewed and analyzed by the Cross Cancer Institute Department of Radiology Bone Density Group, as well as the study investigators. Patient height and weight were also recorded at the time of DXA scan. DXA scans were obtained following enrolment to study (pre-operatively for patients undergoing neoadjuvant systemic therapy or post-operatively for patients under adjuvant treatment) and then repeated approximately 1 year from the date of the first DXA scan. To correct for differences in the time between baseline and end-of-study DXA scans, changes in body composition are reported as percent change from baseline per month. To correct for the possible confounding effect of breast cancer surgery on total body mass and composition, we specifically report alterations in lean and fat body mass relative to the lower extremities, except where the analysis of android to gynoid fat distribution is concerned.

### Stool microbial composition analysis

Stool samples were collected pre- and post-systemic treatment and frozen at −80°C. DXA scans were performed within 30 days of stool collection. Sample timing methodology is detailed in the Supplementary Information. Stool microbial DNA was extracted using the FastDNA Spin kit for Feces (MP Biomedicals) for subsequent whole-genome shotgun sequencing. Metagenome libraries were constructed using the Nextera XT (Illumina) protocol. Libraries were sequenced in an Illumina MiSeq using a paired-end 300-cycle protocol. Taxonomic classification of sequences was conducted with Kraken against a customized database that included full-genome sequences of bacteria and the human genome assembly GRCh38 [20]. Re-estimation of bacterial abundance was carried out with Bracken.

### Proteomic analysis

Serial blood samples were collected in parallel with stool samples. After blood samples were collected, EDTA plasma samples were stored at −80 °C prior to en masse analysis. None of the samples were thawed and refrozen before analysis. EDTA plasma samples were analyzed by Olink Proteomics AB (Uppsala, Sweden). Using PEA technology, levels of 92 inflammation-related protein biomarkers were measured as described previously [21]. Details are available in Supplementary Information.

### Measurement of fecal calprotectin

Samples were analyzed using the Buhlmann Quantum Blue Calprotectin Assay (Schonenbuch, Switzerland) in combination with the Buhlmann Quantum Blue Reader, a quantitative point-of-care test using lateral flow assay technology. The assay is designed for the selective measurement of the calprotectin antigen by sandwich immunoassay, and the reader quantitatively measures the signal intensity by the lateral flow. Samples were read with 1 of 2 reader systems: a low-range kit with a detectable range from 30 to 300 mg/g, or a high-range kit with a detectable range between 300 and 1800 mg/g. For all samples, they were initially read using the high-range kit, and if the values were below the 300 mg/g cut-off level then the samples were reanalyzed with the low-range kit.

### Statistical analysis

Patient and disease characteristics are presented using descriptive statistics. A paired *t* test was performed to compare expression between time points in various treatment groups. ANOVA testing was used for multivariate analyses. Statistical tests were one or two-sided, as indicated within figure legends, with *P*-value of less than 0.05 considered statistically significant. Relative abundance of bacterial taxa among groups was subjected to linear discriminant analysis (LDA) effect size (LEfSe), which is a method for high-dimensional class comparison and was developed specifically for metagenomics data [22]. To evaluate associations between microbiome abundance and clinical metadata we used the statistical framework microbiome multivariable association with linear models (MaAsLin) [23].

## Results

### Patient characteristics

Forty-three participants were screened and 40 participants were enrolled within this study. For the three patients not enrolled to study, two were excluded because they did not meet enrolment criteria, and the third declined participation during the screening process. Patient baseline characteristics are presented in Table 1. Our study included patients who, in addition to surgery, received systemic cytotoxic chemotherapy only (*n* = 10), systemic endocrine therapy only (*n* = 8), chemotherapy followed by endocrine therapy (*n* = 18), or surgery alone (*n* =4). Given the small number of patients who did not receive any systemic therapy as an adjuvant treatment to surgery, these patients were not analyzed as a separate study group, but were instead included in the analysis of non-chemotherapy treated patients.

**Table 1:** Study population and patient demographics

	Number of patients (***n*** = 40)
**Mean patient age (range)**	56 (29–79)
**Surgical procedure**	
Segmentectomy	12
Mastectomy	28
**Tumor histology**	
Ductal carcinoma	39
Lobular carcinoma	1
**Tumor histological grade**	
1	3
2	17
3	20
**Tumor biomarker status**	
ER/PR positive	32
HER2 positive	7
Triple-negative	7
**Disease stage**	
1A/1B	18
2A/2B	16
3A/3B/3C	6
**Treatment intent with systemic therapy**	
Adjuvant therapy following surgical resection	28
Neoadjuvant prior to surgical resection	8
None	4
**Systemic therapy**	
*Chemotherapy only*	9
TC^a^	5
FEC-D^b^	3
TAC^c^	1
*Chemotherapy followed by endocrine therapy*	19
TC followed by TAM^d^	12
FEC-D followed by TAM or AI^e^	7
*Endocrine therapy alone*	8
TAM	7
AI	1

^a^Docetaxel/cyclophosphamide; ^b^5-fluorouracil/epirubicin/cyclophosphamide + docetaxel; ^c^docetaxel/adriamycin/cyclophosphamide; ^d^tamoxifen; ^e^aromatase inhibitor

### Weight and body composition change

**Fig. 1:**
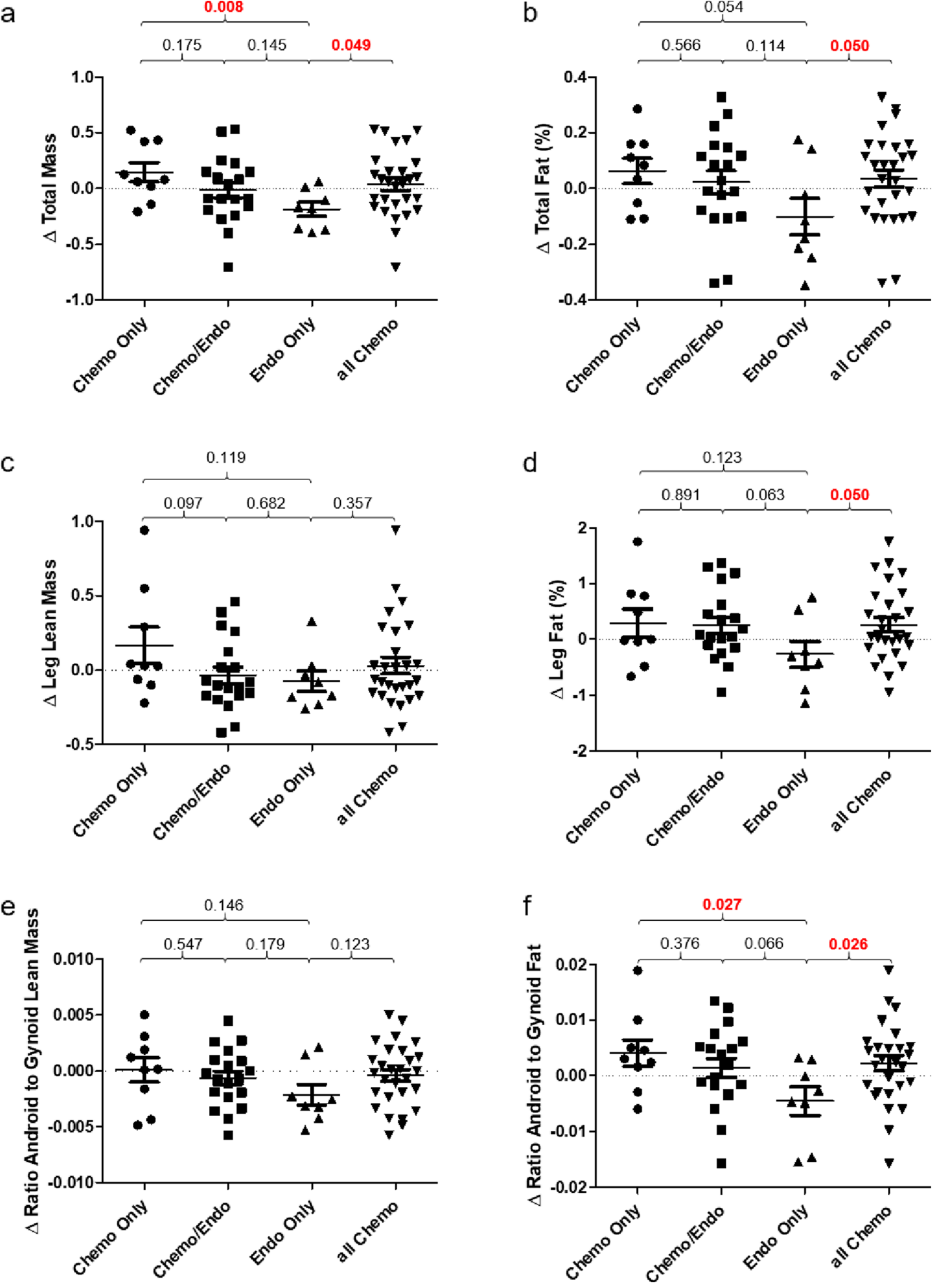
Ratio change in anthropometric measures between patient treatment groups. **a**–**f** Ratio change in DXA determined mass and fat percentages at the end of study normalized to time per month from the start of the study: **a** total body mass, **b** total body fat, **c** leg lean mass, **d** leg fat, **e** android/gynoid lean mass, and **f** android/gynoid fat. *P*-values are displayed above the graphs and significant differences between treatment groups are indicated in red

Figure 1 illustrates changes in body weight (a), total body fat (b), leg lean mass (c), leg fat (d), the ratio between android to gynoid lean mass (e), and the ratio between android to gynoid fat (f) from baseline to one year. Patients treated only with chemotherapy showed increased weight gain as measured by a 0.15% total mass per month ratio increase, as compared to those treated only with endocrine therapy who showed a mean loss of 0.19% total mass per month (Fig. 1a, *P* = 0.008). A significant increase was also seen in the ratio of total mass per month between android to gynoid fat with a ratio of −0.0045 in endocrine-only-treated patients, and a ratio of 0.0041 in chemotherapy-only-treated patients (Fig. 1f, *P* = 0.027). However, we did not observe a significant change in total lean body mass associated with treatment with chemotherapy (Fig. 1e).

**Fig. 2:**
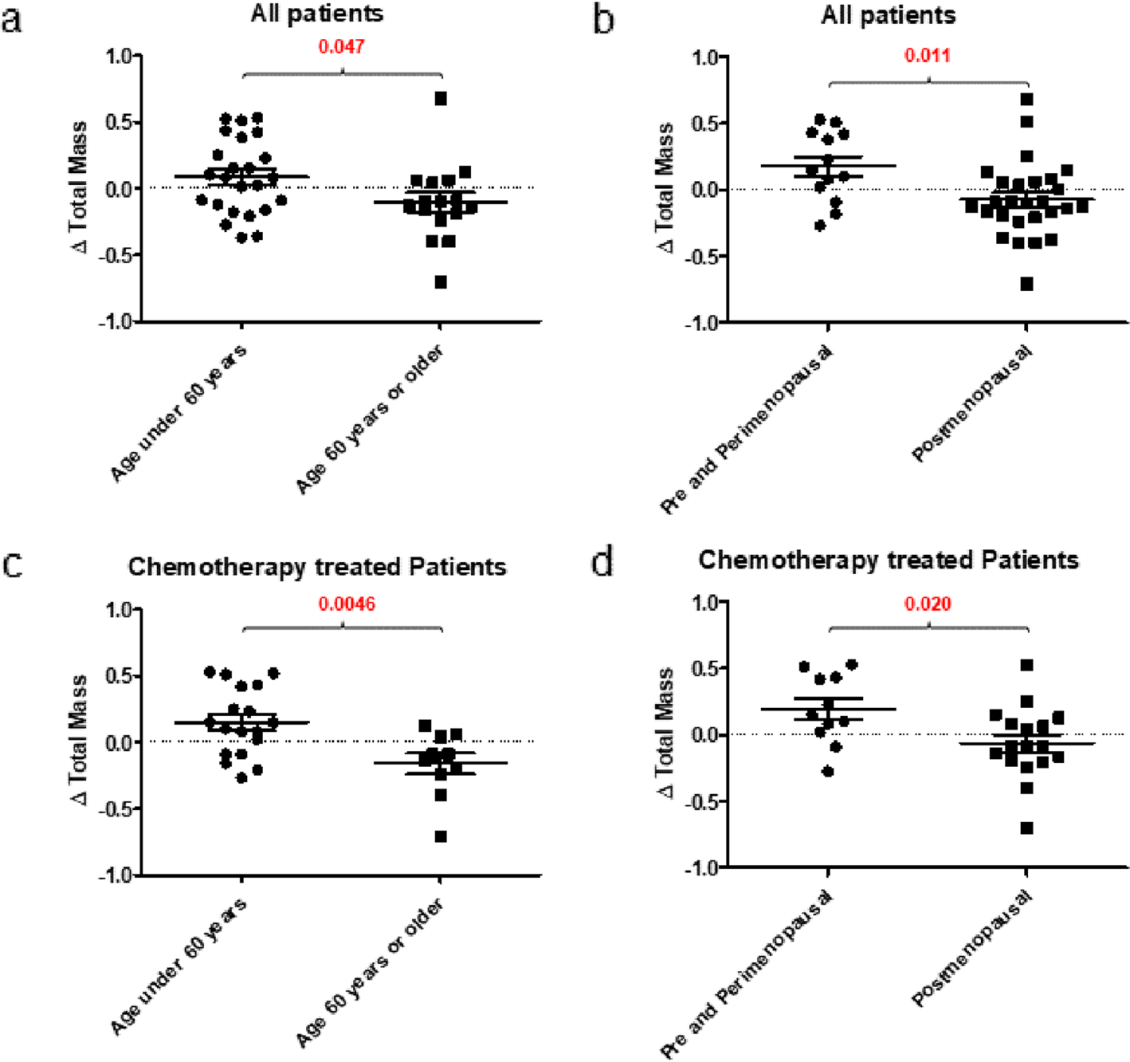
Ratio change total mass of all patients and chemotherapy-treated patients by age and menopausal status. **a**–**d** Ratio change in DXA determined total mass at the end of study normalized to time per month from the start of the study: **a** by age in the whole patient population, **b** by menopause status in the whole population, **c** by age in those treated with chemotherapy, **d** by menopause status in those treated with chemotherapy. *P*-values are displayed above the graphs and significant differences between patient populations are indicated in red

Among our patient cohort treated with chemotherapy, 18 were younger than 60 years of age, and 10 were older than 60 years of age. Furthermore, 11 patients were pre-menopausal, while 17 were post-menopausal. We observed a relative increase in weight gain in younger, pre-menopausal patients, a finding that was consistent across our entire patient cohort (Fig. 2a), but one which was especially pronounced in patients receiving chemotherapy (Fig. 2c, d). Examining the entire patient cohort, those younger than 60 years of age gained weight relative to their baseline with a total mass per month increase of 0.085, while those 60 years of age or older demonstrated a relative reduction in weight with ratio decrease of −0.11 (*P* = 0.047). The same observation was made when patients were stratified based on their menopausal status. Pre-menopausal patients demonstrated a significant increase in weight with a ratio change of total mass per month of 0.18 versus their post-menopausal counterparts who show a ratio decrease to −0.074 (*P* = 0.011). Specifically examining weight gain in patients treated with chemotherapy the same observation was made, as pre-menopausal patients younger than 60 showed a relative increase in weight when compared to older (ratio change 0.15 vs. −0.16; *P* = 0.0046), or pre-menopausal (ratio change 0.19 vs. −0.064; *P* = 0.020) women.

### Baseline differences in gut microbial flora as a function of body habitus

Given the variability of baseline body habitus among patients enrolled in the study, we first analyzed pre-treatment baseline microbial composition as a function of body mass index (BMI). Patients were classified as normal weight (*n* = 14), overweight (*n* = 10), or obese (*n* = 16), using BMI cutoffs of <25, 25–30, and >30, respectively. Among these patient groups, only an *Eggerthella* sp. showed a significant difference with a negative association with obesity (data not shown), in keeping with previously published reports [24]. We also analyzed the Firmicutes to Bacteroidetes ratio (F/B) and found no significant baseline differences between patients classified as normal weight, and overweight or obese. Normal weight patients had an F/B of 5.233, while patients classified as overweight or obese demonstrated ratios of 6.563 and 5.822, respectively (*P* =0.2532).

### Chemotherapy-dependent metagenomic alterations within the gut microbiome

We next analyzed the impact of chemotherapy on the gut microbiome. Among patients treated with chemotherapy, we observed significant alterations in microbial species. Table 2 provides a list of individual bacterial species in which a significant change in abundance was detected following treatment with chemotherapy and/or endocrine therapy. In total, 47 individual species of bacteria were altered in their relative richness following treatment with chemotherapy; 31 species were reduced in relative abundance while 16 species were increased. Following treatment with endocrine therapy, our analysis revealed 16 bacterial species were significantly changed in relative abundance (11 species were increased and 5 species were decreased).

**Table 2:** Significant alterations within the microbiome following treatment with chemotherapy

Bacteria	Phylum	Paired difference pre and post chemotherapy	Chemotherapy paired ***t*** test ***P***-value	Paired difference pre and post endocrine	Endocine paired ***t*** test ***P***-value
*Eggerthella_lenta*	Actinobacteria	**−2213.24**^a^	0.0249	1705.20	0.1979
*Ruminococcus*_sp_5_1_39BFAA	Firmicutes	**−1991.78**	0.0467	1709.31	0.0566
*Coprococcus_eutactus*	Firmicutes	**−1408.99**	0.0390	531.38	0.6447
*Eubacterium_limosum*	Firmicutes	**−1345.83**	0.0196	379.88	0.7068
*Dorea_longicatena*	Firmicutes	**−539.86**	0.0330	179.41	0.6605
*Eubacterium_ventriosum*	Firmicutes	**−387.33**	0.0405	4.52	0.7699
*Eubacterium_rectale*	Firmicutes	**−385.76**	0.0489	**101.99**	0.0477
*Ruminococcus_obeum*	Firmicutes	**−335.56**	0.0203	−66.32	0.5297
*Eubacterium_hallii*	Firmicutes	**−321.98**	0.0222	86.83	0.4395
*Ruminococcus_bromii*	Firmicutes	**−310.81**	0.0381	140.90	0.1650
*Ruminococcus_lactaris*	Firmicutes	**−262.81**	0.0443	**175.58**	0.0408
*Dorea_formicigenerans*	Firmicutes	**−205.70**	0.0243	−6.20	0.9005
*Faecalibacterium_prausnitzii*	Firmicutes	**−197.37**	0.0244	**107.52**	0.0273
*Bifidobacterium_longum*	Actinobacteria	**−145.04**	0.0475	43.72	0.4658
*Pseudoflavonifractor_capillosus*	Firmicutes	**−142.70**	0.0135	7.25	0.7236
*Lachnospiraceae*_bacterium_2_1_58FAA	Firmicutes	**−123.40**	0.0240	51.78	0.4341
*Clostridium_nexile*	Firmicutes	**−103.86**	0.0287	25.12	0.4864
*Anaerostipes_hadrus*	Firmicutes	**−98.86**	0.0328	**68.35**	0.0359
*Holdemania_filiformis*	Firmicutes	**−96.75**	0.0130	18.54	0.6626
*Lachnospiraceae*_bacterium_1_4_56FAA	Firmicutes	**−93.18**	0.0084	−20.09	0.6968
*Oscillibacter*_sp_KLE_1745	Firmicutes	**−92.72**	0.0113	0.57	0.8362
*Clostridium_difficile*	Firmicutes	**−90.63**	0.0037	**62.75**	0.0379
*Erysipelotrichaceae*_bacterium_6_1_45	Firmicutes	**−88.91**	0.0279	20.27	0.6309
*Bifidobacterium_pseudocatenulatum*	Actinobacteria	**−88.90**	0.0090	76.46	0.2646
*Eubacterium_ramulus*	Firmicutes	**−87.12**	0.0272	−1.07	0.9814
*Anaerotruncus_colihominis*	Firmicutes	**−76.52**	0.0263	**125.05**	0.0335
*Bacteroides_fragilis*	Bacteroidetes	**−72.47**	0.0398	**−617.32**	0.0338
*Lachnospiraceae*_bacterium_1_1_57FAA	Firmicutes	**−66.19**	0.0224	−17.52	0.7283
*Clostridium_clostridioforme*	Firmicutes	**−54.17**	0.0158	41.60	0.2429
*Anaerostipes_caccae*	Firmicutes	**−51.00**	0.0485	71.93	0.2595
*Clostridium_citroniae*	Firmicutes	**−48.79**	0.0116	29.33	0.2610
*Clostridium_bolteae*	Firmicutes	**53.47**	0.0243	−5.21	0.3687
*Clostridium_asparagiforme*	Firmicutes	**69.40**	0.0471	35.90	0.4319
*Bacteroides_coprocola*	Bacteroidetes	**73.82**	0.0325	111.77	0.1583
*Bacteroides_clarus*	Bacteroidetes	**78.20**	0.0339	54.37	0.5244
*Ruminococcus_gnavus*	Firmicutes	**98.14**	0.0405	1.24	0.3913
*Clostridium_hathewayi*	Firmicutes	**99.15**	0.0445	−58.06	0.3724
*Bacteroides_caccae*	Bacteroidetes	**101.28**	0.0422	36.38	0.6709
*Bacteroides_barnesiae*	Bacteroidetes	**104.08**	0.0410	24.30	0.8197
*Bacteroides_ovatus*	Bacteroidetes	**106.44**	0.0466	−21.12	0.6440
*Bacteroides_uniformis*	Bacteroidetes	**113.81**	0.0313	**197.86**	0.0284
*Bacteroides_dorei*	Bacteroidetes	**123.27**	0.0439	−0.92	0.9919
*Roseburia_intestinalis*	Firmicutes	**162.79**	0.0119	28.78	0.1766
*Ruminococcus_torques*	Firmicutes	**167.92**	0.0322	**116.36**	0.0123
*Bacteroides_finegoldii*	Bacteroidetes	**195.39**	0.0330	**116.89**	0.0382
*Clostridium_scindens*	Firmicutes	**268.08**	0.0411	−58.14	0.6986
*Bacteroides_pectinophilus*	Bacteroidetes	**383.29**	0.0298	−93.49	0.4232
*Bacteroides_intestinalis*	Bacteroidetes	50.43	0.7363	**−243.28**	0.0430
*Clostridium*_sp_ATCC_BAA_442	Firmicutes	134.56	0.2143	**−234.69**	0.0210
*Bifidobacterium_adolescentis*	Actinobacteria	115.36	0.3852	**−158.22**	0.0241
*Bacteroides_vulgatus*	Bacteroidetes	14.74	0.7399	**−78.56**	0.0368
*Clostridium_symbiosum*	Firmicutes	12.47	0.6194	**108.89**	0.0333
*Ruminococcus_callidus*	Firmicutes	−80.75	0.3205	**199.02**	0.0322

^a^Significant paired differences are indicated by boldface type

At the species level interesting differences in relative abundance were observed between chemotherapy and endocrine therapy-treated patient groups. For instance, within the group of organisms that were altered in abundance, more species were reduced in relative abundance following treatment with chemotherapy, as opposed to endocrine therapy where the opposite was observed. In our analysis, two-thirds of the bacterial species which were affected in relative abundance after treatment with chemotherapy were reduced in abundance. Conversely, among those patients treated with only endocrine therapy, nearly 70% of the species altered in abundance following treatment were increased relative to pre-treatment. Looking specifically at the change in relative abundance among the chemotherapy-treated patients, further taxonomic analysis of the change in microbial abundance is revealing. We observed a predominant reduction within the Firmicutes phylum, whereas phyla Bacteroidetes demonstrated a relative increase in abundance; conversely, among the endocrine-treated patients an increase in Firmicutes organisms was observed. Among the Firmicutes bacteria affected by treatment with chemotherapy, more than 80% are reduced in relative abundance following treatment with chemotherapy, while treatment with endocrine therapy resulted in a relative increase in abundance of nine out of ten affected Firmicutes species. Among Bacteroidetes organisms affected by treatment with chemotherapy, 90% of the species were increased in relative abundance following treatment with chemotherapy, a result not seen in endocrine therapy patients.

### Linear discriminant effect of pre- and post-chemotherapy microbial signatures

**Fig. 3:**
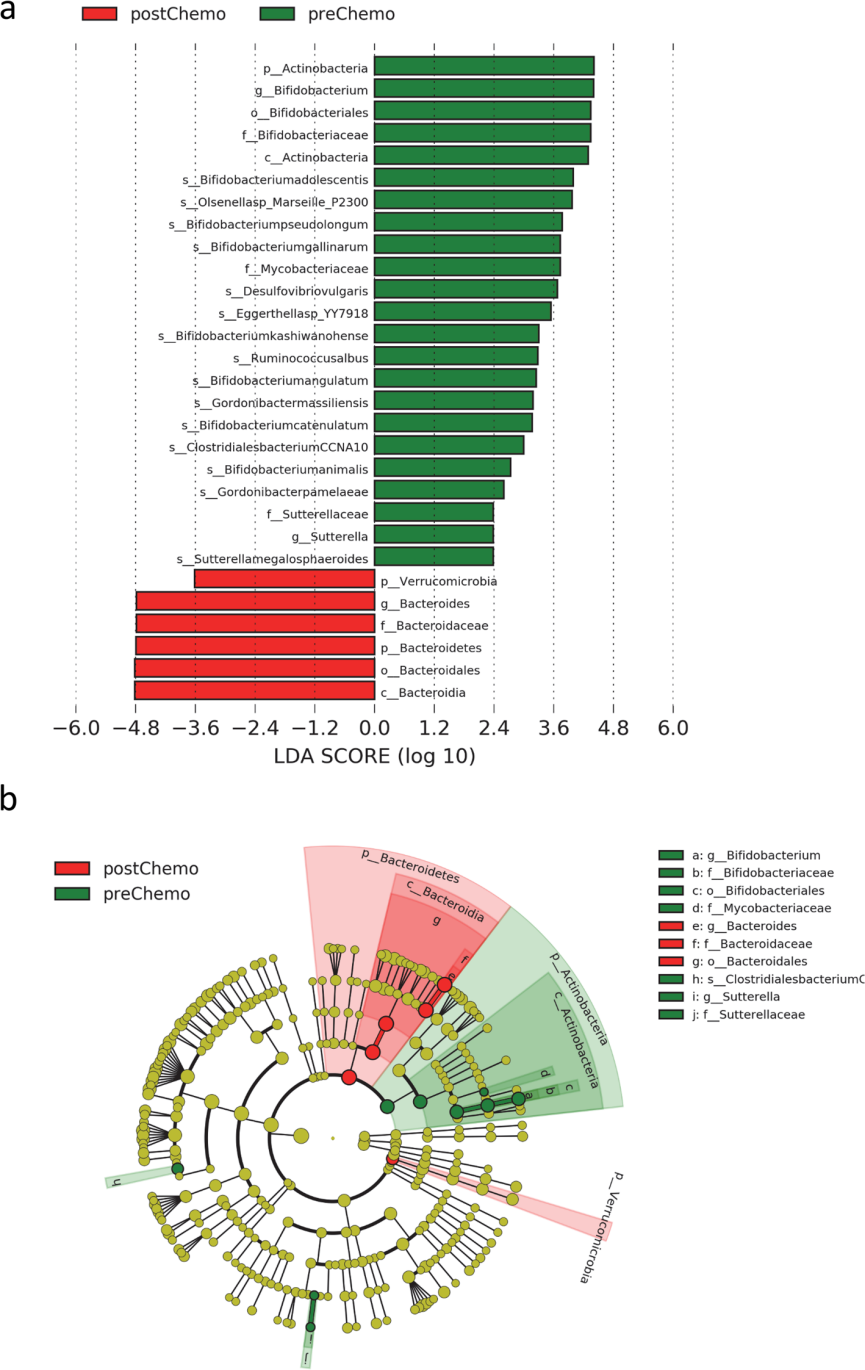
Linear discriminant effect analysis of chemotherapy-associated microbial alterations. **a** LDA was conducted with LEfSe software. Bacterial cell counts were used as input and normalization, and estimation of size effects and plotting were conducted with LEfSe. Comparisons with a *P*-value <0.05 and a LDA score >2 were considered different. **b** Cladogram derived from LEfSe analysis including genera in the innermost circle up to phyla in the outermost circle. Diameter of each node is proportional to the abundance of each taxon. In **a** and **b**, taxa that were found to be more abundant in the pre-chemotherapy group are colored green, while taxa that were found more abundant in the post-chemotherapy group are colored red

We next conducted a linear discriminant effect size analysis (LEfSe) to determine which bacterial taxa most contributed to the chemotherapy-associated perturbation of the gut microbiome. With our analysis, we found no significant differences between the microbiome signature in patients’ pre- and post-endocrine therapy, and as further control, no significant differences were observed in patients when pre- and post-operative pre-systemic therapy samples were analyzed. However, we observed significant differences between stool sample analysis before and after treatment with chemotherapy. In total, 23 and 6 species of bacteria were significantly associated with pre- and post-chemotherapy microbial signatures, respectively (Fig. 3). Analysis by LEfSe demonstrated a relative homogeneity in the post-chemotherapy microbiome signature when compared with samples obtained prior to chemotherapy. Phylum representation appeared more diverse in the pre-chemotherapy samples, whereas in the post-chemotherapy sample there appeared to be a relative abundance of Bacteroidetes.

### A pro-inflammatory proteomic signature is observed following treatment with chemotherapy

An association between intestinal microbial dysbiosis and gut inflammation has been well documented in disease states such as inflammatory bowel disease [15, 25]. We analyzed patient inflammatory biomarkers to evaluate a potential pro-inflammatory response that could perhaps link chemotherapy-induced microbial changes with anthropometric change. As described in Table 3, we detected increases in pro-inflammatory cytokines/chemokines following treatment with chemotherapy. These same changes were not observed within the group of patients who only received endocrine therapy as an adjuvant treatment to surgery, indicating the phenomenon was indeed related to treatment with chemotherapy. In fact, the inflammatory signature in patients receiving endocrine therapy was the opposite of that seen with chemotherapy treatment as a general reduction in inflammatory biomarkers was observed in these participants.

**Table 3:** Treatment-associated protein signatures that were statistically significant between pre- and post-treatment samples

	Post-chemotherapy relative expression			Post-endocrine therapy relative expression		
Protein	Mean paired difference	Percent difference	Paired ***t*** test	Mean paired difference	Percent difference	Paired ***t*** test
IL-17A	0.3166	29%	**0.0132**^a^	0.0650	6%	0.6717
IL6	0.5848	14%	**0.0038**	−0.1904	−4%	0.4658
CDCP1	0.4438	11%	**0.0040**	−0.3879	−9%	**0.0085**
CXCL10	0.8327	10%	**0.0077**	−0.5239	−6%	0.0579
CX3CL1	0.5470	9%	**0.0001**	−0.5142	−8%	**0.0007**
ADA	0.3710	8%	**0.0003**	−0.2177	−5%	*0.0470*
CCL20	0.4305	8%	**0.0042**	−0.1810	−3%	0.4132
FGF-21	0.5143	8%	**0.0057**	−0.7357	−10%	0.0503
IL18	0.6073	7%	**0.0005**	−0.3188	−4%	0.0777
CCL3	0.3421	6%	**0.0073**	−0.2909	−5%	**0.0168**
IL8	0.3384	6%	**0.0101**	−0.3491	−5%	0.0720
MCP-1	0.5978	6%	**0.0000**	−0.2949	−3%	0.0538
Flt3L	0.5379	6%	**0.0001**	0.0005	0%	0.9965
PD-L1	0.2038	5%	**0.0499**	−0.1732	−4%	0.0561
IL-10RB	0.3209	4%	**0.0004**	−0.2137	−3%	**0.0193**
CCL23	0.4187	4%	**0.0005**	−0.4554	−4%	**0.0038**
uPA	0.3538	3%	**0.0009**	−0.4534	−4%	**0.0008**
IL-12B	0.1478	3%	0.2428	−0.3075	−6%	**0.0375**
CSF-1	0.2376	3%	**0.0014**	−0.0919	−1%	0.3834
CST5	0.1767	3%	**0.0437**	−0.0878	−1%	0.3355
MCP-3	0.0441	3%	0.8297	−0.3090	−15%	**0.0330**
CD40	0.2415	2%	**0.0168**	−0.2054	−2%	**0.0166**
MCP-4	0.0852	2%	0.3817	−0.3250	−8%	**0.0286**
VEGFA	0.1733	2%	**0.0293**	−0.1070	−1%	0.3012
TNFRSF9	0.0322	0%	0.7571	−0.3080	−5%	**0.0021**
TRAIL	0.0018	0%	0.9841	−0.2848	−3%	**0.0273**
TWEAK	−0.0133	0%	0.8668	−0.2412	−2%	**0.0154**
SCF	−0.0461	0%	0.5764	−0.3934	−4%	**0.0029**
CD5	−0.0493	−1%	0.5766	−0.2315	−4%	**0.0193**
TRANCE	−0.1874	−4%	0.0568	−0.3970	−9%	**0.0078**
EN-RAGE	−0.9922	−28%	**0.0051**	−0.3380	−12%	0.1530

^a^Significant paired differences are indicated by boldface type

Of note, chemotherapy-treated patients showed a sharp rise in circulating interleukin-17 levels. We also detected an increase in circulating interleukin-6, interleukin-8, and interleukin-18 after treatment with chemotherapy but not after endocrine therapy. We further observed significant increases in the levels of circulating chemokines including CCL3, CCL23, CCL7, CCL20, CXCL10, and fractaline (CX3CL1), all of which have been associated with interferon-dependent inflammatory signaling.

We also assayed for changes in specific cytokines and chemokines which are known to play a role in metabolic disease. We observed a significant increase following chemotherapy treatment in the serum concentrations of fibroblast growth factor-21 (FGF21) as well as the monocyte chemotactic protein-1 (MCP1) chemokine.

### Colonic inflammation occurs following treatment with chemotherapy

**Fig. 4:**
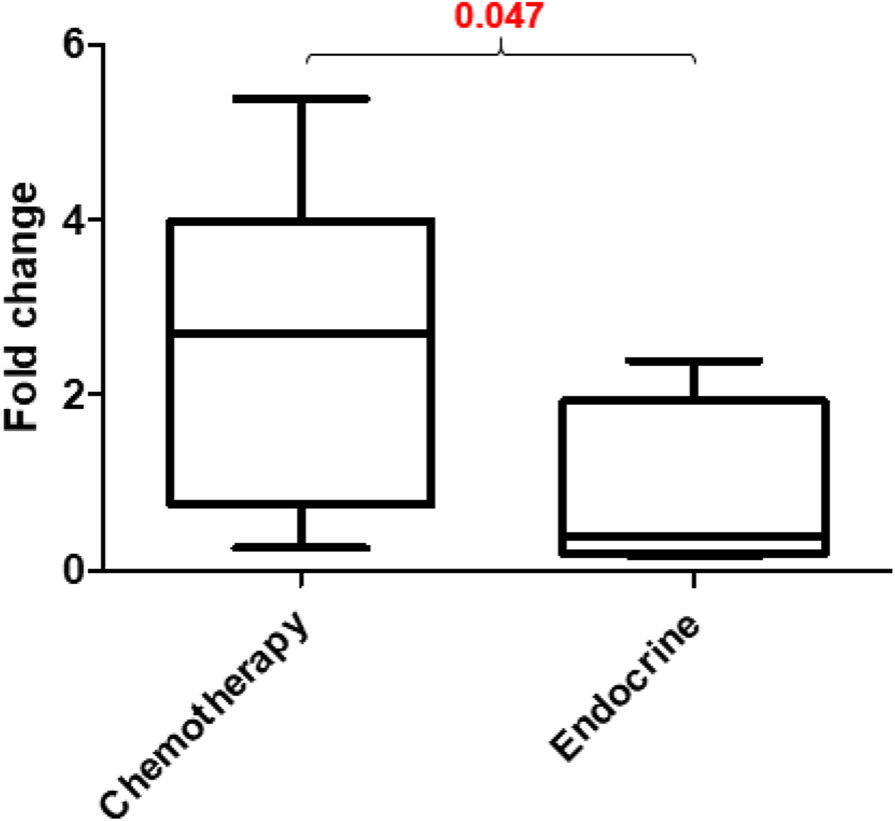
Fecal calprotectin levels after chemotherapy and endocrine treatment. Sandwich immunoassay for quantitative fecal calprotectin levels reveals a significant fold-increase from baseline in patients following adjuvant chemotherapy when compared to calprotectin levels in patients treated with adjuvant endocrine therapy (*P* = 0.047). Stool samples used for analysis were the same as those used for microbial analysis (Figs. 3 and 4)

Treatment with chemotherapy can induce severe gastrointestinal tract toxicity but the underlying mechanisms are not fully understood. Calprotectin is an antimicrobial protein that is elevated in patients with active IBD, and whose presence is indicative of neutrophil migration into the lumen of the intestine [26]. We measured fecal calprotectin levels as a biomarker for intestinal inflammation in patients treated with chemotherapy and endocrine therapy to compare baseline pre-treatment levels with post-treatment levels. As shown in Fig. 4, a significant fold-increase over baseline is observed in patients who were treated with adjuvant chemotherapy, but not in those patients who only received treatment with endocrine therapy. Patients who received chemotherapy demonstrated a 2.646-fold increase in measured calprotectin levels after treatment, while patients who were treated with endocrine therapy saw no such increase (fold-increase 0.8350, *P =* 0.0473).

## Discussion

In this prospective and longitudinal study of patients with surgically resected early-stage breast cancer, we observed alterations within the gut microbiome following treatment with chemotherapy, as well as significant weight gain and an increase in body fat. Furthermore, the perturbation of the microbiome was accompanied by the development of an inflammatory response that may link chemotherapy-dependent alterations within the gut microbiome with our reported anthropometric changes.

Obesity has been implicated in the development of breast cancer [27] and weight gain after treatment may impact the risk of disease recurrence and death [28]. Furthermore, the impact of weight gain during survivorship is a significant public health concern given the high prevalence of breast cancer. Quite notably, prior studies have revealed that the proportion of overweight or obese individuals increases from less than 50% to 67% when pre- and post-cancer diagnosis BMI are compared [29].

Whereas much of the available evidence to suggest an association between treatment with chemotherapy and weight gain is retrospective or anecdotal, we present prospectively collected data and furthermore we conducted DXA scans, the gold standard for body composition analysis both pre- and post-treatment. We also collected and analyzed stool and blood samples in conjunction with DXA studies, which allowed us to test the hypothesis that chemotherapy-dependent alterations in gut microbial ecology impacts host metabolism. Our study confirms earlier reports of weight gain after treatment with chemotherapy for early-stage breast cancer, but in addition, our study was designed to include appropriate control groups to permit a more extensive interpretation of our data. In particular, we included collection of data from patients treated only with endocrine therapy as adjuvant treatment following surgery, a group of patients we believe comprise the optimal comparator group. Strikingly, within this comparator group we observed none of the findings we report within our chemotherapy-treated cohort of patients.

The use of DXA for body composition analyses within our study was important for the added information it provides beyond weight gain or loss. Android fat deposition is a sensitive predictor of subsequent metabolic disease [30] and more recently, an inverse association between intestinal microbial diversity and android fat distribution was reported [31]. With our results we demonstrate the use of adjuvant cytotoxic chemotherapy, but not adjuvant endocrine therapy, is associated with significant weight gain and increased adiposity in patients with early-stage breast cancer. More specifically, our results demonstrate a significant increase in the ratio of android to gynoid fat deposition in chemotherapy-treated patients that was not seen in patients treated with only endocrine therapy. Furthermore, we demonstrate stability of lean body mass across our entire patient cohort, including those patients treated with and without chemotherapy, suggesting chemotherapy-associated body composition changes may be isolated to increased adiposity. It has been suggested that chemotherapy-associated weight gain during treatment for early-stage breast cancer may be transient [5], but if in fact body composition (i.e., an increase in body-fat percentage) and not just weight is altered during treatment with chemotherapy, the long-term health consequences may be significant even if an individual returns to their pre-chemotherapy weight. We did not record change in body composition or weight beyond approximately one year following enrolment to study, but we believe our end-of-study findings of sustained patient weight gain, increased body-fat proportion, and increased android to gynoid body fat distribution imply a significant risk for the development of long-term health consequences.

With respect to the impact of gut microbes on obesity, a few central themes appear consistent within the literature. First, a reduction with respect to bacterial diversity may contribute to the development of obesity as well as a host of additional diseases, perhaps most notably IBD [15]. And second, apart from inter-species variation, the compositional shift of the microbiota at higher hierarchical levels demonstrates an association with disease in human studies and animal models [32, 33]. We observed a significant treatment effect on microbial composition within the gut following treatment with chemotherapy, but not in women who received only endocrine therapy as adjuvant treatment. In our analysis, treatment with chemotherapy predominantly resulted in a reduction in microbial abundance, whereas treatment with endocrine therapy had a dissimilar effect. Phylum representation appeared more diverse in the pre-chemotherapy samples, whereas in the post-chemotherapy sample there appeared to be a relative abundance of Bacteroidetes. This observation suggests that the effect of chemotherapy on the gut microbiome may be to reduce the diversity of the population as opposed to a simple, pan-organism bactericidal effect.

The prospective nature of our study allowed for a comparison of intra-patient variation with respect to microbiota, as we were able to analyze pre- and post-treatment samples from the patients within our study. This is important as the composition of the gut microbiota is known to be dynamic [34], even within an individual, and without pre- and post-treatment sampling it would be difficult to determine whether differences between patients groups (e.g., chemotherapy and endocrine therapy-treated) truly relates to a treatment effect.

As with prior studies, we observed greater post-chemotherapy weight gain among younger patients [5, 35], although given the small number of patients within this subgroup specific caution should be exercised when interpreting our data. We stratified our patient cohort both by age and menopausal status and observed increased weight gain among patients younger than 60 years of age, and also in the group of patients who were pre-menopausal. The relationship between estrogen, the gut microbiome, and health and disease is an emerging area of study, and the estrobolome, an aggregate of enteric bacterial genes whose products are capable of metabolizing estrogens, has been described [36]. Biliary excretion of estrogen and its metabolites is a well-known phenomenon [37] and indeed, estrogen metabolites in conjugated form may be recovered from feces [38, 39]. Furthermore, antibiotic exposure in pre-menopausal women has been shown to increase estrogen excretion [40], possibly through perturbation of the gut microbiome and a corresponding reduction in the deconjugation of non-absorbable metabolites of the hormone. The effect of estrogens on metabolism is well known, with estrogen deficiency contributing to the development of obesity and the metabolic syndrome [41, 42]. As is the case with antimicrobial therapy, perturbation of the microbiome associated with chemotherapy may result in a reduction in the re-absorption of estrogen metabolites from the gastrointestinal tract. Thus, the relative increase in weight gain observed in younger patients may be due in part to an anti-estrogenic effect. Conversely, the pleiotropic effects of estrogen are reduced with aging through multiple mechanisms, including a reduction in the level of circulating hormone but also through a reduction in estrogen receptor expression via transcriptional and epigenetic mechanisms [43, 44], which may explain why older, postmenopausal patients exhibit less weight gain versus their younger, pre-menopausal counterparts.

At present, we do not fully understand the complex mechanisms by which perturbation of gut microbiota may impact human health and disease, but others have postulated these changes may be mediated through systemic inflammation (recently reviewed by Cox et al [45]). After recognizing that treatment with chemotherapy appears to reduce microbial diversity and abundance within the gut in a manner similar to that seen in active IBD, we measured circulating cytokines and chemokines among our patient cohort, as well as fecal calprotectin levels. For patients who received treatment with chemotherapy an increase in fecal calprotectin levels as well as circulating pro-inflammatory mediators was observed, but a similar response was not seen among patients who received treatment with endocrine therapy after breast cancer surgery; in fact, the opposite appeared true. We observed a robust inflammatory response in chemotherapy-treated patients characterized by more than a 2.5-fold increase in fecal calprotectin levels, as well as increased levels of interleukin-6, interleukin-8, interleukin-17, and interleukin-18. We also discovered an increase in circulating chemokine levels following treatment with chemotherapy, and while a component of this response may indicate a homeostatic response to chemotherapy-induced cytopenias, when considered as a whole the picture is more consistent with a pro-inflammatory response similar to that which may be seen in the setting of IBD. The relationship between gut microbiota and inflammation seen with inflammatory bowel disease is a complex one and evidence suggests that perturbation of the microbiome may lead to the development of aberrant inflammation rather than inflammation leading to perturbation of the microbiome. Further study will be required to determine whether the same process occurs following treatment with chemotherapy, however, we believe such a scenario may provide a mechanistic link between treatment with chemotherapy, resultant intestinal microbial dysbiosis, and the subsequent occurrence of weight gain and metabolic disease seen within these patients.

Within our patient cohort, weight gain after treatment with chemotherapy was associated with increased FGF-21 and MCP1 levels. Within the gut FGF-21, a hepatokine secreted by the liver functions as a potent activator of glucose uptake in adipocytes [46]. FGF-21 is elevated in obese subjects and is independently associated with the development of type 2 diabetes [47]. MCP1 is known to confer resistance to insulin signaling [48], and we think that the increase after treatment with chemotherapy seen with both cytokines may be of particular significance within the context of weight gain and body compositional changes. Interestingly, the increase of circulating MCP-1 within our chemotherapy-treated patients is of additional interest when considered within the broader literature as MCP-1 is known to play a key pathogenic role in the development of immune-mediated illness including colitis [49, 50].

The increase in measured chemokines including chemokine ligand 23 (CCL23) may also represent a mechanism by which chemotherapy results in systemic inflammation. CCL23 is a chemokine with potent chemoattractant properties for resting T-lymphocytes [51]. CCL23-dependent signaling has been implicated in several inflammatory disease states including rheumatoid arthritis [52] and IBD [53]. Similarly, our observed upregulation of interleukin-18 (IL-18), a member of the IL-1 family of cytokines, may translate treatment with chemotherapy to inflammation. IL-18 plays a major role in the induction of interferon-gamma signaling, and an increase in circulating IL-18 has been demonstrated in numerous inflammatory disease states including metabolic syndromes, psoriasis, and IBD [54]. Although we are limited by a relatively small sample size, our study informs the possibility of the potential development of gut inflammation in subsets of chemotherapy-treated patients.

Previous studies established that chemotherapy is associated with harmful anthropometric change, including weight gain, which is a known risk factor for poor patient outcomes in survivorship after treatment for early-stage breast cancer. In this study, we link cytotoxic chemotherapy with perturbation of the gut microbiome and development of a robust inflammatory response with resultant weight and body compositional changes.

We acknowledge the extensive recent work performed by Terrisse et al. [19], in which the authors also demonstrate patient weight gain following treatment with chemotherapy. However, the alterations within the gut microbiome within the study by Terrisse would appear opposite to those that we observed within our prospective study. Why treatment with cytotoxic therapy would increase the diversity of bacterial populations within the gut is unclear, but it seems likely that any change within the gut microbiome after treatment with chemotherapy would be transient, and thus the difference between the results of our two studies may reflect a difference in the timing of sample collection. Our analysis of the effect of chemotherapy on the composition of the gut microbiome was made on samples collected just before or immediately following a patient’s final cycle of cytotoxic therapy — whereas if samples were collected at too distant a time following cessation of treatment the true chemotherapy effect may have been missed.

We also note the recent work of Uzan-Yulzari et al., in which evidence is supplied to indicate baseline microbiome characteristics may predict weight gain and metabolic derangement after treatment with chemotherapy [55]. We find this hypothesis intriguing and believe it merits further investigation. This work is complementary to our own; however, our study design allows us to demonstrate that treatment with chemotherapy also alters the composition of the gut microbiome (in addition to any baseline changes which may be relevant) in association with these anthropometric changes.

Strengths of our study include a priori data and biosample collection. Our matched-cohort study design allowed for the enrolment of 40 individuals with early-stage breast cancer, divided between those patients who received chemotherapy after curative-intent surgery, and those who would only receive endocrine therapy following surgery, thus including within our analysis a population of patients whom we believe to represent the optimal control group for studying chemotherapy-dependent microbiome and body compositional change. Our study represents a comparatively large analysis with respect to characterization of the microbiota in the setting of chemotherapy, and indeed cancer in general, but nonetheless, given the complexity of the gut microbiome our sample size still limits the interpretation and generalizability of our data. In addition, prospective collection of biomarkers specific for metabolic disease would have added value to the study, and future projects will include prospective data collection over a longer duration, as well as prospective evaluation of glycaemic control, blood pressure, and lipid indices following treatment with chemotherapy.

## Conclusions

Biological mechanisms by which post-chemotherapy alterations within the gut microbiota could lead to weight gain are yet to be elucidated. However, it has been postulated that a systemic inflammatory response may occur due to a microbial shift, which could lead to the development of obesity or disease. We sought to connect alterations within the gut microbiota with the development of systemic inflammation as a consequence of cytotoxic chemotherapy. Notably, we show that treatment with chemotherapy, but not endocrine therapy, results in a transient rise in circulating pro-inflammatory cytokines and chemokines, as well as a significant increase in fecal calprotectin levels, which indeed indicates a connection between post-chemotherapy gut microbiota change and inflammation. Moreover, this illuminates a potential mechanism by which chemotherapy could result in weight gain in breast cancer survivorship, and a possible target of future treatments designed to improve long-term patient outcomes.

## Supplementary Information

**Additional file 1: Supplementary Methods.** Methodological detail on patient stool sample collection and proteomic analysis.

## Data Availability

The datasets used and/or analyzed during the current study are available from the corresponding author on reasonable request.

## Abbreviations

IBD: Inflammatory bowel disease
DNA: Deoxyribonucleic acid
RNA: Ribonucleic acid
DXA: Dual-energy X-ray absorptiometry
LDA: Linear discriminant analysis
LEfSe: Linear discriminant analysis effect size
MaAsLin: Microbiome multivariable association with linear models
BMI: Body mass index
FGF21: Fibroblast growth factor-21
MCP1: Monocyte chemotactic protein-1
CCL23: Chemokine ligand 23
IL-18: Interleukin-18
