# Supplementary material for: Chemotherapy-induced weight gain in early-stage breast cancer: a prospective matched cohort study reveals associations with inflammation and gut dysbiosis
Source: BMC Med. 2023 May 11;21:178. doi: 10.1186/s12916-023-02751-8 (PMC10173591; doi:10.1186/s12916-023-02751-8)
Supplement: Supplementary file 1 — Additional file 1: Supplementary Methods. Methodological detail on patient stool sample collection and proteomic analysis. [file 12916_2023_2751_MOESM1_ESM.pdf]

Chemotherapy-induced inflammation links gut dysbiosis with metabolism and weight gain in early-stage breast cancer: a prospective matched cohort study

John Walker et al.

Supplementary Methods

Sample collection timing

For patients who received chemotherapy as neoadjuvant treatment prior to surgery, stool samples were collected at baseline (prior to beginning treatment with chemotherapy), following the end of chemotherapy (pre-operatively), and at approximately one-year from enrolment to study. For patients who received only endocrine therapy as adjuvant treatment to surgery, stool samples were collected at baseline (pre-operatively), then again in the post-operative period (prior to beginning endocrine therapy), and at approximately one-year from enrolment to study. For patients who received adjuvant chemotherapy following surgery, stool samples were collected at baseline (pre-operatively), then again in the post-operative period (prior to beginning chemotherapy), following the end of chemotherapy, and at approximately one-year from enrolment to study.

Proteomic analysis

EDTA plasma samples were analyzed by Olink Proteomics AB (Uppsala, Sweden). Using PEA technology, levels of 92 inflammation-related protein biomarkers were measured. Pairs of oligonucleotide-labeled antibody probes bind to their targeted protein, and if the two probes are brought in close proximity the oligonucleotides will hybridize in a pair-wise manner. The addition of a DNA polymerase leads to a proximity-dependent DNA polymerization event, generating a unique PCR target sequence. The resulting DNA sequence is subsequently detected and quantified using a microfluidic

real-time PCR instrument (Biomark HD, Fluidigm Corporation, CA, USA). Data is quality controlled and normalized using an internal extension control and an inter-plate control to adjust for intra- and inter-run variation. The final assay read-out is presented in Normalized Protein eXpression (NPX) values, which is an arbitrary unit on a log2-scale proportional to protein expression. Proteins were excluded from analysis if >20% of individual measurements were below the lower limit of detection. When <20% was below lower limit of detection, missing values were replaced by the limit of detection divided by two. All assay validation data for the proteins in the inflammation panel (detection limits, intra- and inter-assay precision data, accuracy, *et cetera*) are available on manufacturer's website (www.olink.com).
